# Supplementary material for: Planktonic microbial signatures of sinking particle export in the open ocean’s interior
Source: Nat Commun. 2023 Nov 7;14:7177. doi: 10.1038/s41467-023-42909-9 (PMC10630432; doi:10.1038/s41467-023-42909-9)
Supplement: Supplementary file 1 — Supplementary Information [file 41467_2023_42909_MOESM1_ESM.pdf]

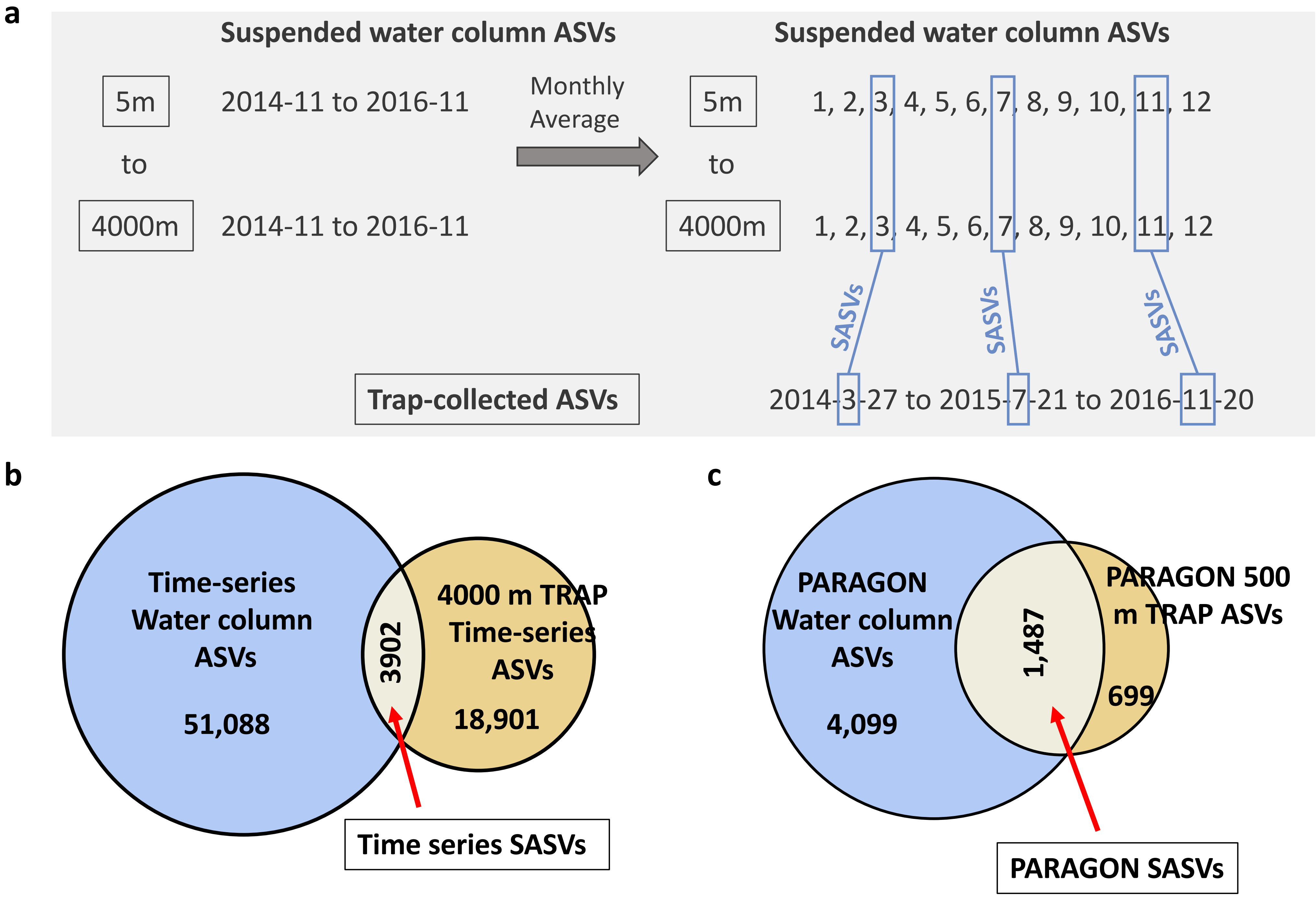

Supplementary Fig. 1. a) Diagram showing the workflow used to detect time-series SASVs shared between suspended planktonic and sediment trap microbial assemblages. b) The yields of unique ASVs for time-series depth-resolved water column samples (2014-2016) and bottom-moored 4000 m sediment traps (2014-2016), and the corresponding numbers of 4000m trap-shared SASVs. c) Yield of the total ASVs for water column samples and 500 m sediment traps during the PARAGON cruise, and the corresponding number of total 500 m trap-shared SASVs.

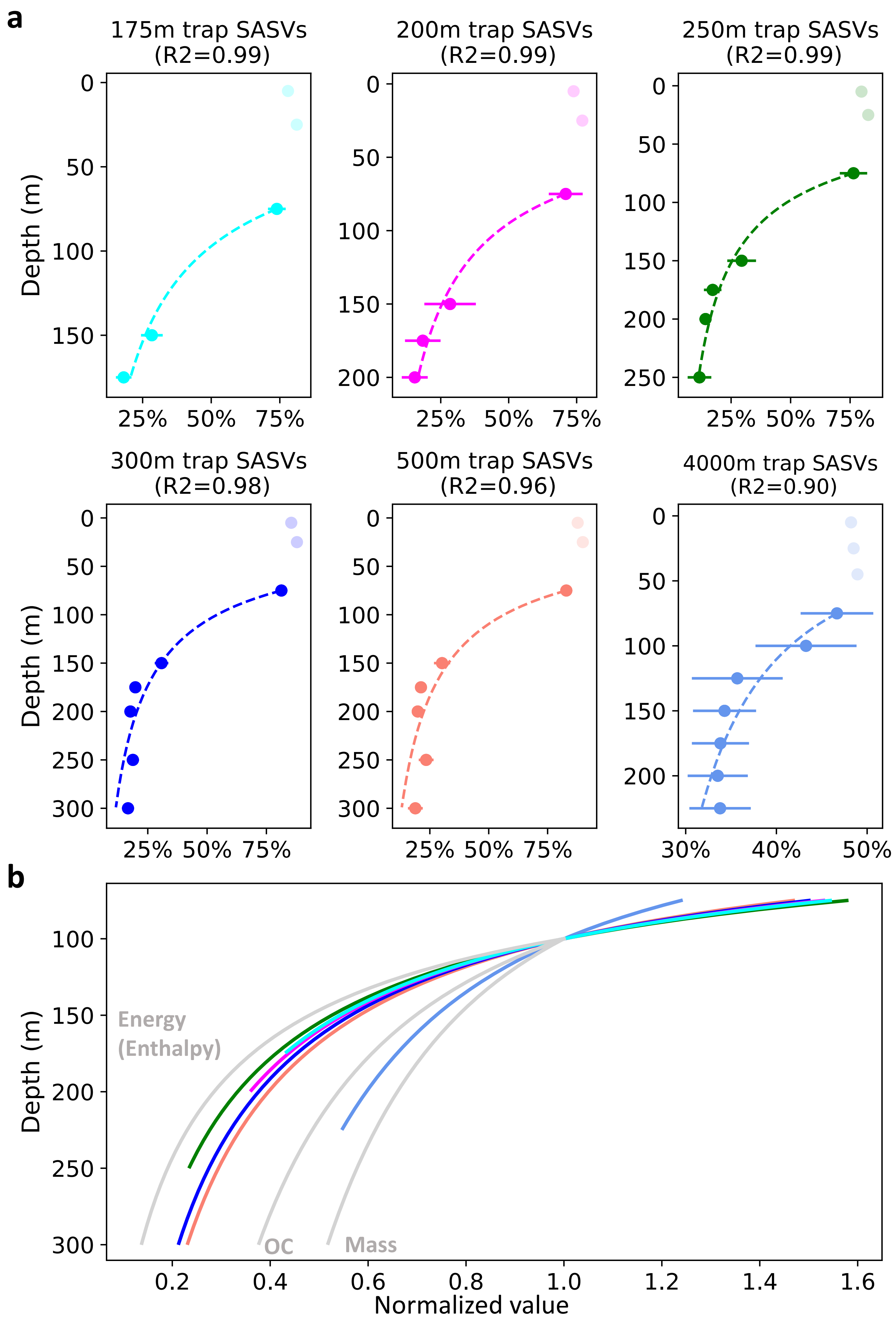

Supplementary Fig. 2. a) Water column averaged relative abundances of PARAGON cruise and Station ALOHA time-series SASVs in upper water column, and their respective power-law curves (Supplementary Data 1). R^2 values are shown for each curve. The horizontal bars represent standard deviation. Where bars are not visible, they are smaller and therefore obscured by the individual data points. b) Schematic representation of power-law curves from 2a, and those previously reported for energy, organic carbon and mass from Grabowski et al.[1] (grey) are shown. Colors correspond to those shown in 2a, above.

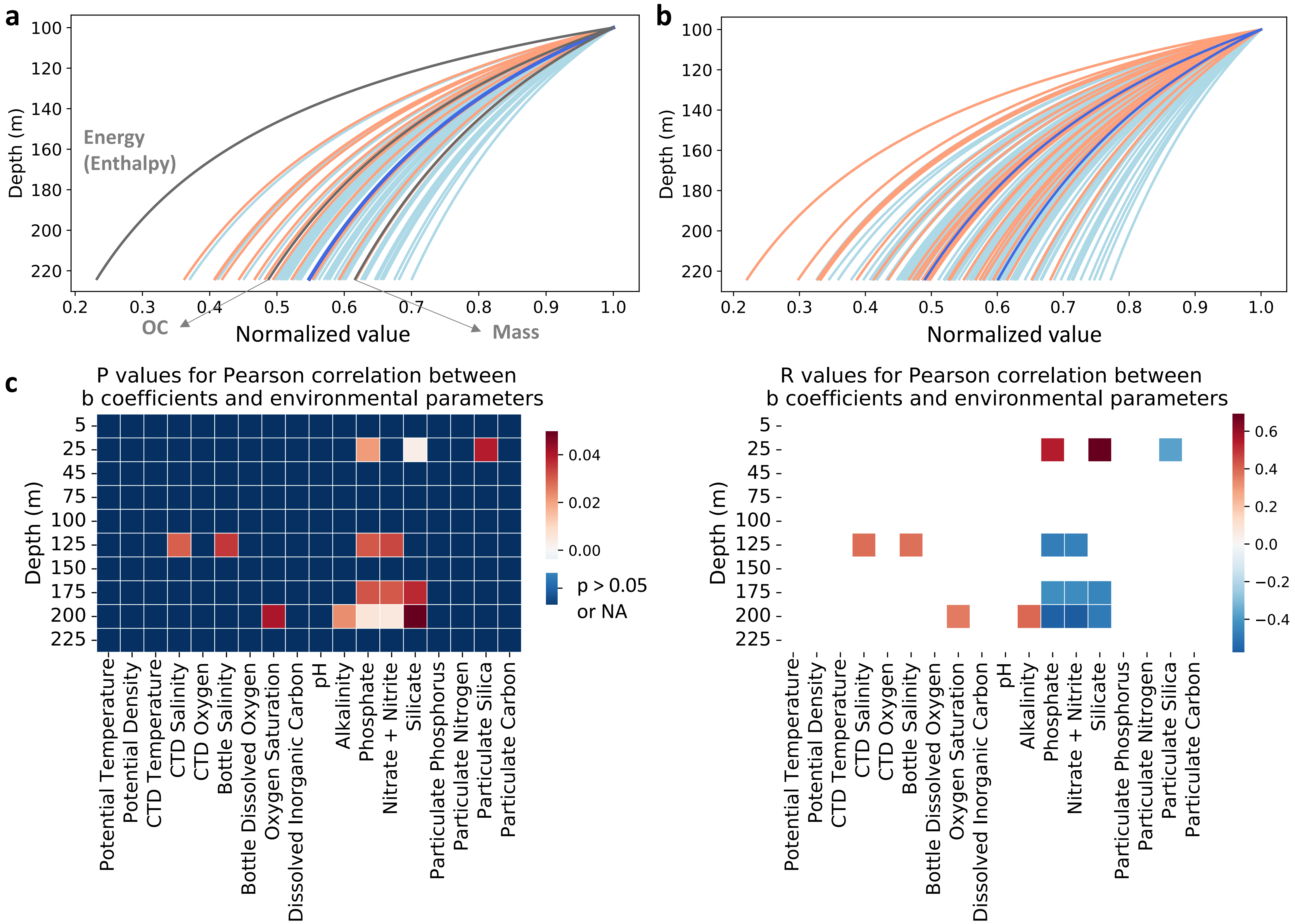

Supplementary Fig. 3. a) Schematic representation of power-law curves for water column relative abundance of Station ALOHA time-series SASVs from all time points and the average data. The black lines represent previously reported attenuation curves for energy, organic carbon and mass from Grabowski et al.[1]. The light blue represents each time point. The dark blue color represents the average data for all time points. The pink color represents SEP time points. b) Schematic representation of 95% confidence intervals of power-law curves from a. c) P and r values for Pearson correlations between b coefficients from SASV power-law curves and environmental parameters from each depth in the shallower water column. Dark blue in p value heatmap and white color in r value heatmap represent the correlation is not significant ($p > 0.05$) or not available (NA, the data points for environmental parameters are less than five.)

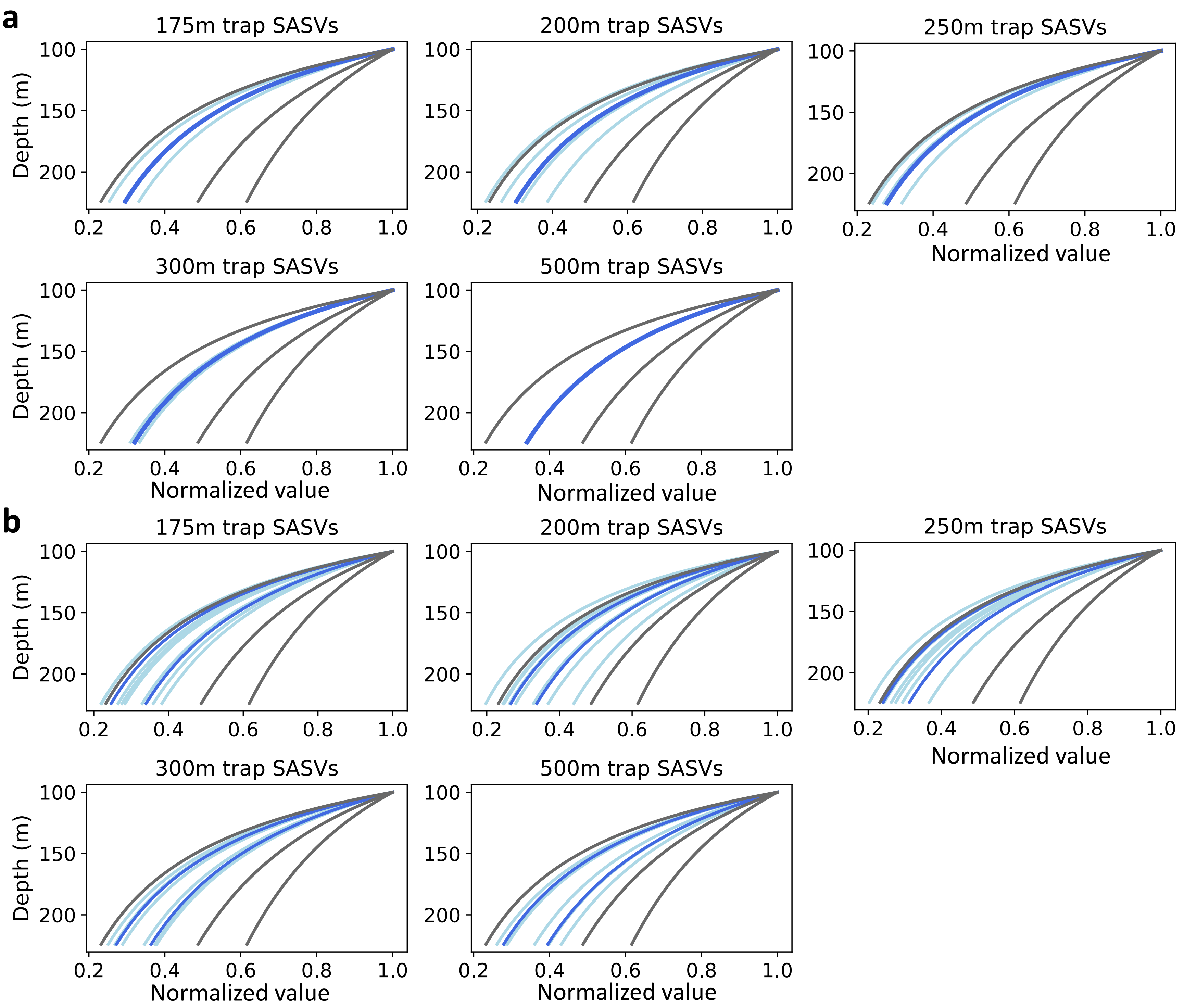

Supplementary Fig. 4. a)Schematic representation of power-law curves for water column relative abundance of PARAGON cruise SASVs from all replicates and the average data collected at each depth. The black lines represent previously reported for attenuation curves for energy, organic carbon and mass from Grabowski et al.[1]. The light blue represents each replicate. The blue color represents the average data of replicates. b) Schematic representation of 95% confidence intervals of power-law curves from a, above.

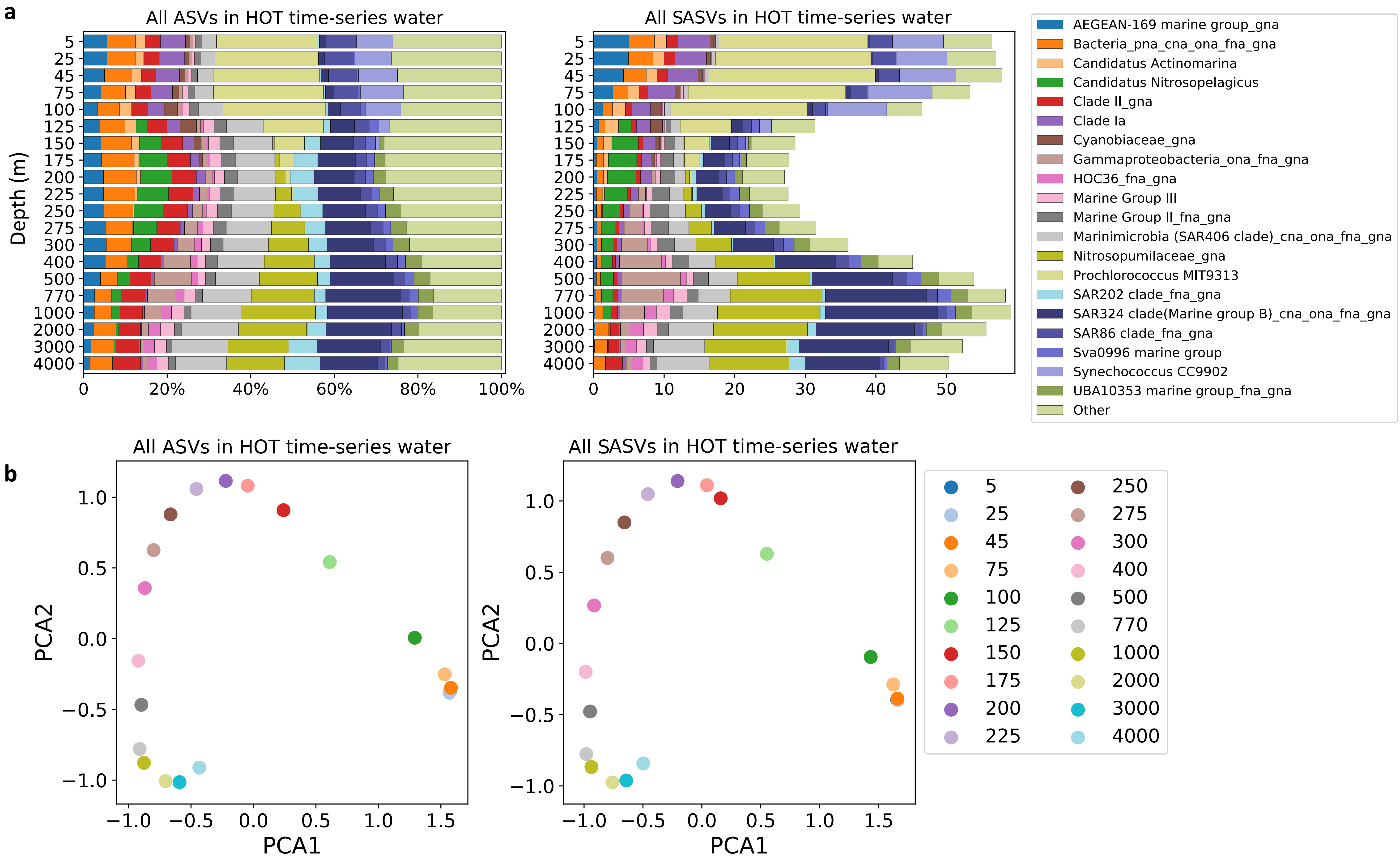

Supplementary Fig. 5. a) Vertical profile of prokaryotic community composition of Station ALOHA time-series ASVs and SASVs in the water column, during all the entire sampling period. b) PCA analysis of time-series ASVs and SASVs in the water column. The "pna" designator indicates that phylum level taxonomy is not available or unknown, as do corresponding indicators for cna (class), ona (order), fna (family) and gna (genus).

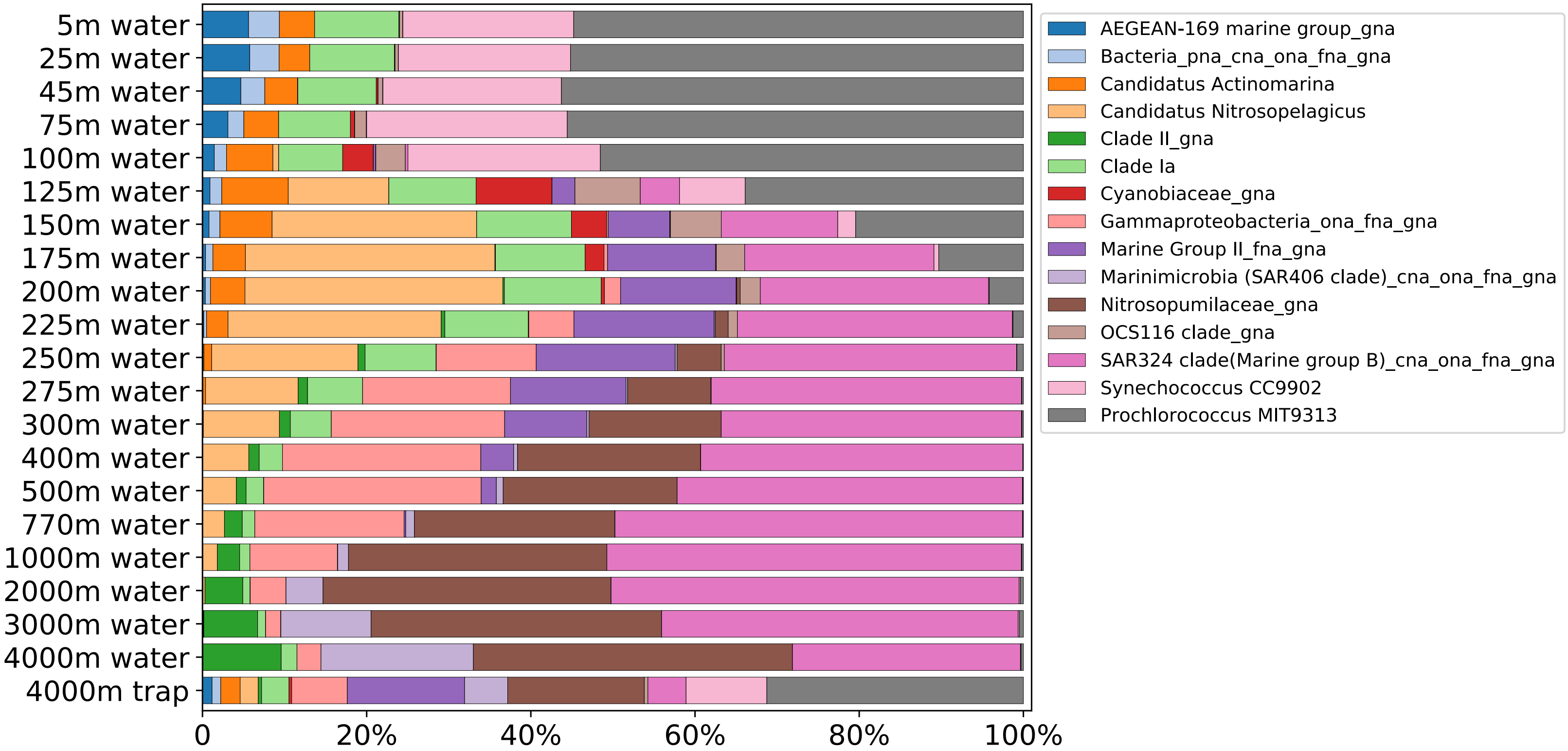

Supplementary Fig. 6. Taxonomic profiles of the top five 4000 m trap SASVs from each depth in terms of water column abundances. Deep water prokaryotes, like SAR406 and Nitrosopumilaceae, were greatly enriched in 4000 m trap particles reaching 4000 m, compared to euphotic zone water column. This supports the in situ contribution of subeuphotic oligotrophic prokaryotes to export of sinking particles.

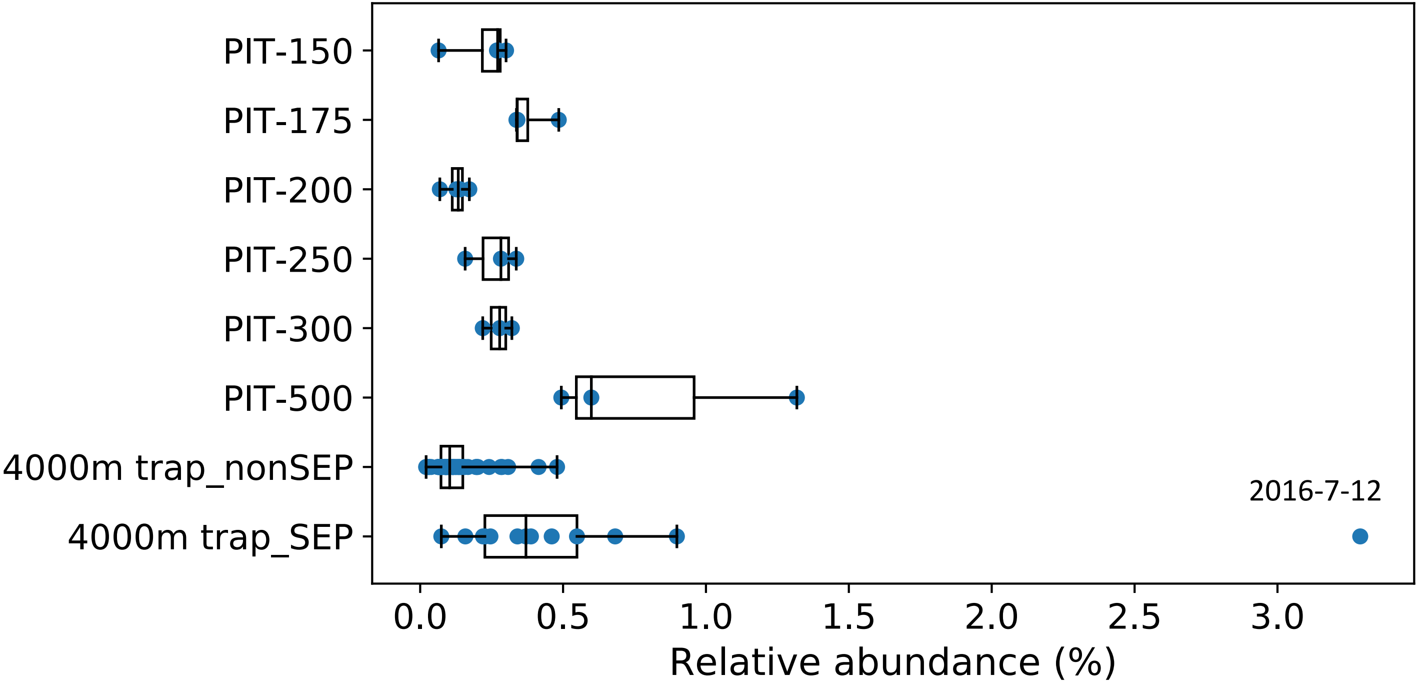

Supplementary Fig. 7. Box plots of Prochlorococcus and Synechococcus SASV relative abundances in sediment traps.

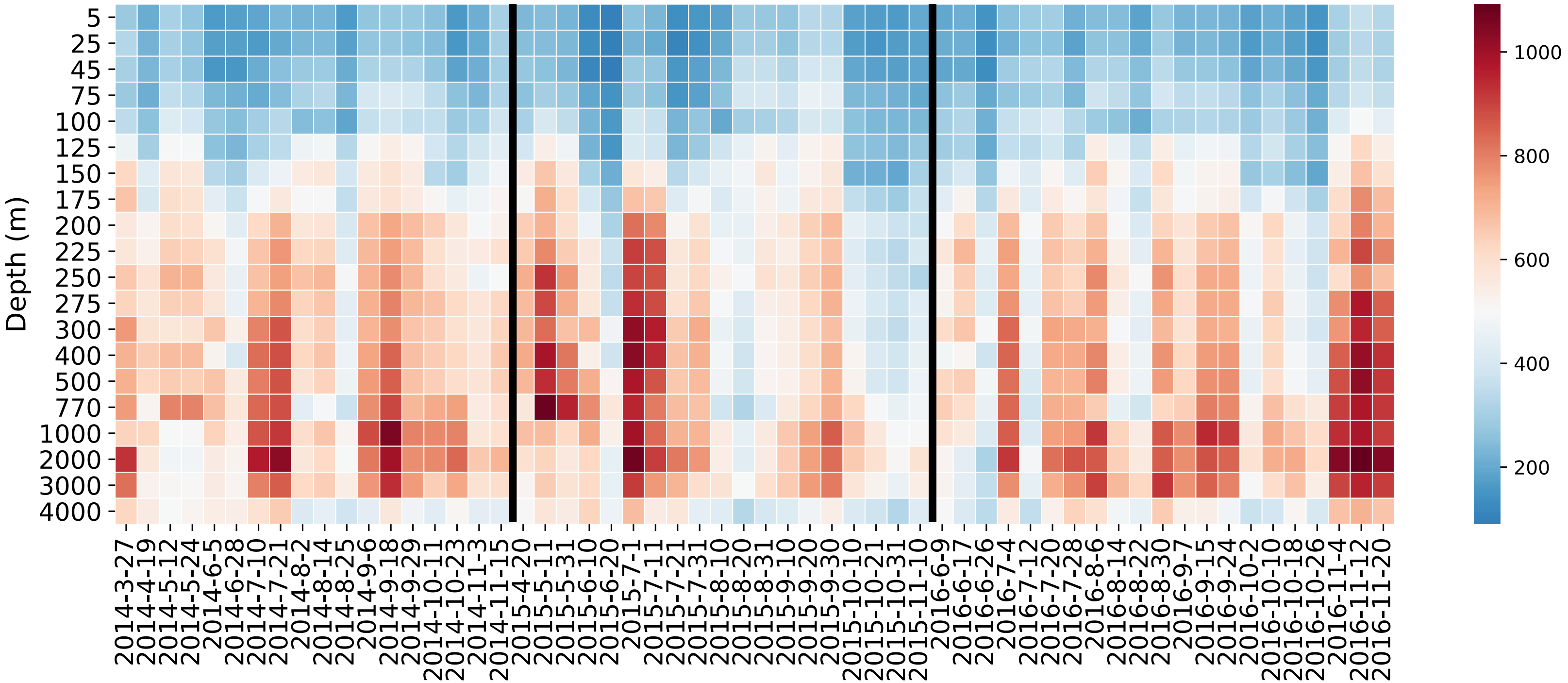

Supplementary Fig. 8. Richness of 4000m trap-shared SASVs over depth and across the time series. The vertical black line represents a break in time between 2014-11-15 and 2015-4-20 and between 2015-11-10 and 2016-6-9.
