# Supplementary material for: Planktonic microbial signatures of sinking particle export in the open ocean’s interior
Source: Nat Commun. 2023 Nov 7;14:7177. doi: 10.1038/s41467-023-42909-9 (PMC10630432; doi:10.1038/s41467-023-42909-9)
Supplement: Supplementary file 3 — Description of Additional Supplementary Files [file 41467_2023_42909_MOESM3_ESM.pdf]

Description of Additional Supplementary Files

File Name: Supplementary Data 1
Description: The water column relative abundances of shallower and deep trap SASVs.

File Name: Supplementary Data 2
Description: Parameters of each power-law curve for the water column relative abundances of sediment trap-shared SASVs from each time point in the shallower depths.

File Name: Supplementary Data 3
Description: The specific SASVs identified in Fig. 3 and their taxonomic affiliations.

File Name: Supplementary Data 4
Description: The trap relative abundances of shallower and deep trap SASVs.

File Name: Supplementary Data 5
Description: The taxonomic affiliations of SASVs identified as enriched in 4000 m traps during SEP compared to non-SEP time periods.

File Name: Supplementary Data 6
Description: The sampling information and metadata of Station ALOHA time-series water samples

File Name: Supplementary Data 7
Description: The sampling information on PARAGON samples
