# Supplementary material for: Planktonic microbial signatures of sinking particle export in the open ocean’s interior
Source: Nat Commun. 2023 Nov 7;14:7177. doi: 10.1038/s41467-023-42909-9 (PMC10630432; doi:10.1038/s41467-023-42909-9)
Supplement: Supplementary file 11 — Reporting Summary [file 41467_2023_42909_MOESM11_ESM.pdf]

nature portfolio

Corresponding author(s): Edward F. DeLong

Last updated by author(s): Oct 15, 2023

Reporting Summary

Nature Portfolio wishes to improve the reproducibility of the work that we publish. This form provides structure for consistency and transparency in reporting. For further information on Nature Portfolio policies, see our Editorial Policies and the Editorial Policy Checklist.

Statistics

For all statistical analyses, confirm that the following items are present in the figure legend, table legend, main text, or Methods section.

n/a	Confirmed	
☐	☒	The exact sample size (*n*) for each experimental group/condition, given as a discrete number and unit of measurement
☐	☒	A statement on whether measurements were taken from distinct samples or whether the same sample was measured repeatedly
☐	☒	The statistical test(s) used AND whether they are one- or two-sided<br>*Only common tests should be described solely by name; describe more complex techniques in the Methods section.*
☒	☐	A description of all covariates tested
☒	☐	A description of any assumptions or corrections, such as tests of normality and adjustment for multiple comparisons
☐	☒	A full description of the statistical parameters including central tendency (e.g. means) or other basic estimates (e.g. regression coefficient) AND variation (e.g. standard deviation) or associated estimates of uncertainty (e.g. confidence intervals)
☐	☒	For null hypothesis testing, the test statistic (e.g. *F*, *t*, *r*) with confidence intervals, effect sizes, degrees of freedom and *P* value noted<br>*Give P values as exact values whenever suitable.*
☒	☐	For Bayesian analysis, information on the choice of priors and Markov chain Monte Carlo settings
☒	☐	For hierarchical and complex designs, identification of the appropriate level for tests and full reporting of outcomes
☒	☐	Estimates of effect sizes (e.g. Cohen's *d*, Pearson's *r*), indicating how they were calculated

Our web collection on statistics for biologists contains articles on many of the points above.

Software and code

Policy information about availability of computer code

Data collection	Illumina MiSeq software was used for intiital sequence data QA
Data analysis	Trimmomatic v0.36, Qiime v1.9.1, DADA2 v1.14.1 and find_peaks(), curve_fit(), scipy.stats.pearsonr(), statistics.stdev() and scipy.stats.ttest_ind() in Python v3.8.1 were used in our data analysis.<br>All code used in this study that is not already publicly available, can be found at: https://github.com/fuyanli121/Codes_SASV-paper_2023.

For manuscripts utilizing custom algorithms or software that are central to the research but not yet described in published literature, software must be made available to editors and reviewers. We strongly encourage code deposition in a community repository (e.g. GitHub). See the Nature Portfolio guidelines for submitting code & software for further information.

Data

Policy information about availability of data

All manuscripts must include a data availability statement. This statement should provide the following information, where applicable:

- Accession codes, unique identifiers, or web links for publicly available datasets
- A description of any restrictions on data availability
- For clinical datasets or third party data, please ensure that the statement adheres to our policy

All 16S rRNA gene amplicon sequences have been deposited NCBIs Sequence Read Archive, under the following accession codes: All SSU rRNA amplicon sequences have been deposited in the NCBI Sequence Read Archive under project number PRJNA482655 (4000 m trap amplicons), PRJNA352737 (Station ALOHA suspended

prokaryote amplicons), and PRJNA966198 (PARAGON expedition particle-associated and suspended prokaryote amplicons). All other metadata are available either in this report and associated Supplementary Tables, or in Boeuf et al28 and Poff et al. 13 (4000 m sediment trap data), Grabowski et al. 5 (attenuation curve data for POC, particle mass or particle energy content), or on the Station ALOHA Hawaii Ocean time-series website (water column oceanographic parameters for suspended ASV time-series collections).

Research involving human participants, their data, or biological material

Policy information about studies with human participants or human data. See also policy information about sex, gender (identity/presentation), and sexual orientation and race, ethnicity and racism.

Reporting on sex and gender	Our research doesn't involve human participants, their data, or biological material
Reporting on race, ethnicity, or other socially relevant groupings	Our research doesn't involve human participants, their data, or biological material
Population characteristics	Our research doesn't involve human participants, their data, or biological material
Recruitment	Our research doesn't involve human participants, their data, or biological material
Ethics oversight	Our research doesn't involve human participants, their data, or biological material

Note that full information on the approval of the study protocol must also be provided in the manuscript.

Field-specific reporting

Please select the one below that is the best fit for your research. If you are not sure, read the appropriate sections before making your selection.

☐ Life sciences ☐ Behavioural & social sciences ☒ Ecological, evolutionary & environmental sciences

For a reference copy of the document with all sections, see nature.com/documents/nr-reporting-summary-flat.pdf

Ecological, evolutionary & environmental sciences study design

All studies must disclose on these points even when the disclosure is negative.

Study description

The connectivity of water column and sinking particles was studied to learn how and where the sinking particles were formed and exported. HOT Station ALOHA time-series sediment trap samples were collected at 4000 m over three years from Mar 2014 through Nov 2016 and their corresponding water column suspended samples were collected from 5 to 4000m over two years from Nov 2014 through Nov 2016. Sinking particles from a special PARAGON expedition in summertime of 2021 were collected at shallow depths (150-500m), and three biological replicates at 250-500m and four biological replicates at 150-200m were applied for each depth. The counterpart water column suspended samples from PARAGON expedition were collected.
No treatment, interaction or design structure was applied in this study.

Research sample

The following research samples were used in our study:
1. Station ALOHA 4000 m time-series sediment trap samples and their corresponding water column suspended samples from 2014 through 2016. The 2014 4000 m sediment trap sample descriptions were also described in Boeuf et al. (2019) and Poff et al. (2021). These samples are meant to study the export of sinking particles in both upper column water and deep water.
2. PARAGON shallow (150-500m) sediment trap samples and their corresponding water column suspended samples in summertime of 2021. These samples are meant to validate the reproducibility of the results observed in the upper water column based on the 4000m time-series trap samples.

Sampling strategy

The sampling strategy is as follows:
1. Station ALOHA 4000 m time-series sequencing sediment trap samples were collected from individual sample cups every 12 days on average; their corresponding water column samples were collected from 5 to 4000m during HOT time-series cruises on an approximately monthly basis. Biweekly or monthly sample collection over three years is sufficient to cover the seasonal change of sinking particle export. The high resolution of depth sampling is sufficient to cover the spatial variation of sinking particle export.

2. 150, 175, 200, 250, 300 and 500 m sediment trap samples were collected , and three biological replicate subsamples at 250-500m and four biological replicate subsamples at 150-200m were prepared. The corresponding water column suspended samples were collected by CTD water sampling during the same time period. The degradation of sinking particles is more intense at shallow depths which were chosen in our study. The sampling strategy is sufficient for reproducibility of 4000 m time-series samples observation in the upper water column.

No sample size calculation was performed.

Data collection

16S rRNA gene amplicon sequence data were collected by Andrew Burger using Illumina MiSeq platform. CTD water column data was collected by HOT team using standard shipboard methods. The hydrographic parameters can be retrieved in https://hahana.soest.hawaii.edu/hot/hot-dogs

Timing and spatial scale	The timing and spatial scale for the sampling is as follows:<br>1. Station ALOHA 4000 m time-series sediment trap samples were collected every 12 days in average from Nov 2014 through Nov 2016. Their corresponding water column suspended samples were collected monthly over two years from Nov 2014 through Nov 2016 at 20 depths of 5, 25, 45, 75, 100, 125, 150, 175, 200, 225, 250, 275, 300, 400, 500, 770, 1000, 2000, 3000, and 4000m.<br>2. PARAGON sediment trap samples were collected in summertime of 2021 at depths of 150, 175, 200, 250, 300 and 500 m and their water column samples were collected at depths of 5, 25, 75, 150, 175, 200, 250, 300, and 500m.
Data exclusions	2015-5-21 4000 m time-series sediment trap sample was excluded because of failed DNA extractions or amplicon library and sequencing
Reproducibility	Replicates, as well and time-series reproducibility from the sediment trap samples and water column suspended samples were used to examine the reproducibility of the observations on the sinking particle export reflected from Station ALOHA time-series samples. The reproducibility of observations is evident and displayed in several of the manuscript figures.
Randomization	Samples were not allocated into groups for the analysis of sinking particle export over depth, which is to show the high resolution of temporal and spatial variations. Because the taxonomic profiles of water column suspended prokaryotic communities were grouped into depth categories, samples for such analysis were grouped into depth horizons: 5 – 75 m (Surface), 100 – 150m (Deep Chlorophyll Maximum, DCM), 175 – 200 m (Lower Euphotic zone), 225 – 500 m (Upper Mesopelagic zone), 770 -1000 m (Lower Mesopelagic zone) and 2000 – 4000 m (Bathypelagic zone).
Blinding	Not Applicable.

Did the study involve field work? ☒ Yes ☐ No

Field work, collection and transport

Field conditions	Samples were collected with the help of the captains and crews of R/V Kilo Moana, R/V Ka'imikai-O-Kanaloa, the HOT program, and the SCOPE team. The hydrographic parameters can be retrieved in https://hahana.soest.hawaii.edu/hot/hot-dogs
Location	The sampling locations are as follows:<br>1. Station ALOHA time-series sediment trap samples were collected at 22° 45'N, 158° 00'W and at 4000 m;<br>2. Station ALOHA time-series water column suspended samples were collected at 22° 45'N, 158° 00'W and at 20 depths of 5, 25, 45, 75, 100, 125, 150, 175, 200, 225, 250, 275, 300, 400, 500, 770, 1000, 2000, 3000, and 4000m.<br>3. PARAGON sediment trap samples were collected at 22.16'N, 156.54'W and at depths of 150, 175, 200, 250, 300 and 500 m<br>4. PARAGON water column suspended samples were collected at 22.16'N, 156.54'W and at depths of 5, 25, 75, 150, 175, 200, 250, 300, and 500m
Access & import/export	Samples were collected in conjunction with the HOT time-series program in US waters. The Station ALOHA was accessible with HOT cruises. Import or export of samples is not applicable in this study.
Disturbance	All trash generated from sample collection was transported to the land for disposal.

Reporting for specific materials, systems and methods

We require information from authors about some types of materials, experimental systems and methods used in many studies. Here, indicate whether each material, system or method listed is relevant to your study. If you are not sure if a list item applies to your research, read the appropriate section before selecting a response.

Materials & experimental systems

n/a	Involved in the study	
☒	☐	Antibodies
☒	☐	Eukaryotic cell lines
☒	☐	Palaeontology and archaeology
☒	☐	Animals and other organisms
☒	☐	Clinical data
☒	☐	Dual use research of concern
☒	☐	Plants

Methods

n/a	Involved in the study	
☒	☐	ChIP-seq
☒	☐	Flow cytometry
☒	☐	MRI-based neuroimaging
